# Evidence-based sleep promotion in acute care from the perspective of nursing staff: a cross-sectional study

**DOI:** 10.1186/s12912-025-04259-3

**Published:** 2026-01-05

**Authors:** Olga Nilsson, Arja Vehkala-Höglund, Linda Gellerstedt

**Affiliations:** 1 Department of Molecular Medicine and Surgery, Karolinska Institutet, Stockholm, Sweden; 2 Department of Vascular Surgery, Karolinska University Hospital, Stockholm, 171 64 Sweden; 3 Department of Clinical Neuroscience, Karolinska Institutet, Stockholm, Sweden; 4 Department of Neurology, Karolinska University Hospital, Huddinge, Stockholm, Sweden; 5 Division of Nursing, Department of Neurobiology, Care Sciences and Society, Karolinska Institutet, Stockholm, Sweden

**Keywords:** Sleep, Educational needs, Nursing, Inpatients, Acute care, Cross-sectional

## Abstract

**Background:**

When hospitalised, patients’ sleep affects their ability to cope with the physiological and psychological strains of illness, and poor sleep negatively affects their recovery. Nursing staff have an important role in promoting patients’ sleep, yet little is known about their knowledge and attitudes towards sleep promotion. The aim was to describe knowledge and values of evidence-based sleep promotion among nursing staff in acute care. A secondary aim was to assess the attention given to sleep promotion and the implementation of a sleep-promoting intervention.

**Methods:**

A cross-sectional cohort study using web-based questionnaires distributed from April to May, 2024. The questionnaires were sent to nurses and nursing assistants at the intervention units (cardiovascular and neurology departments), where an intervention has been introduced. The intervention is designed to enhance the knowledge and competence regarding in-hospital sleep among nursing staff and comprises three parts; a web-based course, evidence-based clinical guidelines and sleep nursing champions who function as sleep advocates at their respective units. Questionnaires were also distributed to control units at the oncology department, where the intervention is yet to be implemented.

**Results:**

In total, 179 questionnaires were completed, 129/389 (33%) from the intervention units and 50/175 (29%) from the control units. Sleep was rated as highly important, and no statistical differences were seen between the groups regarding knowledge of or attention given to sleep promotion, nor the use of non-pharmacological methods to promote sleep. In the intervention group, only 53% were familiar with the web-based course and half of those had completed the course. The clinical guideline was known to 33% of the intervention group, and corresponding numbers regarding the sleep nursing champions were 36%.

**Conclusion:**

Nursing staff are dedicated to sleep promotion and wish to increase their knowledge of non-pharmacological methods to promote sleep during hospitalization. However, the uptake of a sleep-promoting intervention in an acute care setting was limited and the staff did not utilize the available tools and resources. For successful dissemination of evidence-based sleep-promotion, active implementation strategies utilizing the engagement of patients, healthcare staff and policy makers are crucial.

**Trial registration:**

Retrospectively registered at clinicaltrials.gov, registration number NCT07265713.

## Introduction

Restful sleep is a fundamental part of health, essential to cognition, memory, immunity, hormone regulation, metabolism and organ function [1]. While qualitative sleep is beneficial to a person’s health, poor sleep is associated with increased risk of cardiovascular disease [2, 3], pain [4] and physical disability [5]. In recent years, the scientific focus has shifted from sleep disturbances and sleep deficiency to a more holistic perspective including the concept of sleep health, acknowledging genetic, social, environmental, behavioural and healthcare factors affecting a person’s sleep [6]. According to this perspective, the following five dimensions of sleep are most relevant to sleep health: sleep duration, sleep continuity, timing, alertness/sleepiness and satisfaction/quality [6].

Qualitative sleep is particularly important during hospitalisation, and cohesive sleep is a substantial element to patients’ recovery. It has been shown to affect both pain [4], risk of infections and other postoperative complications [7]. At hospitals, there are a number of factors which affect patients’ sleep negatively, both environmental (sound disturbances, light exposure, room sharing, noise of hospital staff) and behavioural (anxiety, pain, worrying about illness) [8]. Patient-reported sleep deteriorates during hospitalization, and sleep dysfunction persists 3 months after discharge [9]. Efforts to maintain sleep health during hospitalisation are therefore pivotal. Historically, pharmacological treatment has been used extensively to enhance in-hospital sleep but these medications are associated with adverse effects and the benefits often do not outweigh the risks [10, 11]. Therefore, non-pharmacological interventions targeting both personal and environmental factors are recommended [8, 12, 13]. Recent systematic reviews conclude that non-pharmacological interventions such as physical sleep aids, relaxation, manual therapy and music may improve inpatient sleep [14–16].

In a cross-sectional study of 2005 hospitalised patients, a majority reported being awakened due to external factors, of which being disturbed by hospital staff was the most common [8]. Despite the importance of sleep for recovery and health, nurses often have a superficial understanding of sleep physiology and sleep-promoting strategies [17, 18]. Lack of evidence-based knowledge hinder nurses to take affective action to improve patients’ sleep [19]. However, studies highlight that nurses are in fact interested in sleep and recognize its importance for hospitalized patients [12, 17]. To address this gap, it is therefore essential to provide nursing staff with the knowledge and tools to promote patients’ sleep during hospitalisation. For this purpose, an intervention was developed to aid nursing staff in adopting evidence-based, non-pharmacological methods to promote sleep. The intervention consists of three components: a web-based educational course, a clinical guideline and the introduction of sleep nursing champions (SNCs) at the intervention units. The aim of the current study was to describe knowledge and values of evidence-based sleep promotion among nursing staff in acute care. A secondary aim was to assess the attention given to sleep promotion and implementation of a sleep-promoting intervention.

## Methods

### Study design

A single-center cross-sectional study design was chosen to capture the local practices and receptivity to the targeted intervention among nursing staff across two departments [20]. The STROBE checklist was followed for the reporting of the study.

### Ethical considerations

The study received approval by the Swedish Ethical Review Authority (Dnr 2023-04670-01). It was conducted according to ethical principles [21] and permission was granted from the units’ nursing managers. A complementary letter including information about the study and its aim was sent together with the link to the web-based survey. Digital consent to participation was collected prior to completion of the questionnaire. Participants were assured that their participation was voluntary, and that only aggregated data would be analysed and communicated. The trial was retrospectively registered at clinicaltrials.gov, registration number NCT07265713.

### Setting

The study was conducted at Karolinska University Hospital (KUH) in Stockholm. The hospital has two physical sites, Huddinge and Solna and recruited participants from both sites. At the hospital, a project team has taken steps to improve patients’ in-hospital sleep by designing and implementing the intervention. In addition to the authors, the project team consisted of one registered nurse and two nursing specialists from the two hospital sites with a special interest in sleep promotion. The intervention comprises a sleep-promoting care bundle consisting of three interlinked components: a web-based course, an evidence-based sleep guideline and the introduction of SNCs. The intervention is reported in accordance with the Template for Intervention Description and Replication (TIDieR) checklist to ensure completeness and quality of the description [22]. The development and implementation of the project was guided by the i-PARiHS framework, which outlines strategies for integrating and implementing evidence into practice [23]. The i-PARiHS framework guided the multilevel, multimodal design of the intervention to enhance uptake of the intervention in the clinical setting.

The intervention aimed to elevate sleep as a key component of nursing care, enhance health care professionals’ knowledge of sleep, and provide support and practical guidance for sleep promoting practices in the hospital. The web-based course was developed by a web design agency that specializes in pedagogical e-learning courses. It contained four chapters with both basic information about sleep physiology and detailed, in-depth texts and cases about sleep during hospitalisation. It takes around 30–40 min to complete and has interactive elements and quizzes to enhance user engagement and learning. The contents of the course were created jointly by the project team in collaboration with a senior e-learning specialist at the design agency. The course was published on the Learning Management System (LMS) platform “Lärtorget” (available at https://lartorget.sll.se/) and access was initially restricted to the staff at the intervention units. The clinical guideline consisted of evidence-based theoretical aspects of sleep-promotion along with practical guidance on pharmacological and non-pharmacological treatment of sleep disturbances during hospitalisation. The guideline was authored by the project team in collaboration with a clinical pharmacist, and based on systematic reviews, existing guidelines and the Swedish electronic medicines compendium [8, 24–27]. It was formatted to provide nursing staff with relevant knowledge based on the patient’s clinical care trajectory, with indexation such as “Upon hospital admission” and “During ward rounds”. It was published online and distributed locally in paper format. The project team also introduced nursing staff to the guideline through workshops at the units. The third component, SNCs, was implemented at the units to promote and encourage sleep promotion at the intervention units and facilitate implementation. This role, referred to as “local champions” according to the i-PARiHS framework, is considered an essential element to promote the implementation of evidence-based practice [20]. The project group recruited SNCs from the intervention units based on the staffs’ own interest in adopting the role at their respective units. The SNCs were trained by the project team through digital webinars and physical meetings, and supplied with relevant scientific literature and a role description. The role description included educating and tutoring their peers, promoting sleep in the daily clinical work, and restructuring the daily, local routines to enhance patients’ sleep. The SNCs did not have allocated time for the role, but undertook the assignment as an integral part of their day-to-day work duties. They were available to their colleagues via telephone, email or physical inquiry during their work shifts. The intervention was implemented at eight units within the heart and vascular theme, including neurological, neurosurgical, thoracic, cardiac or vascular surgery departments. To select appropriate control units, data regarding to average length of stay, as well as age and comorbidity of the admitted patients was extrapolated from the hospital’s data registries and matched with the intervention units. Following this review, four oncological ward units were selected as control units. The project team has promoted the intervention through emails, local meetings at the intervention units, and announcements on the hospital’s internal web page from February, 2023 and throughout the data collection period until May, 2024.

### Data collection

Participants were identified through staff registries at the hospital’s HR unit. An invitation to participate was sent out to all permanently employed registered nurses and nursing assistants via email, along with detailed information about the purpose and scope of the investigation.

The questionnaire used in the study was originally developed by the author LG [28] and was revised to suit the aims of the current study. It contained questions regarding demography, professional role and experience, as well as questions on knowledge about and attitude to sleep promotion. The questionnaire was constructed in two versions, with additional questions regarding the sleep intervention distributed to nursing staff in the intervention units. Inclusion criteria were nurses or nursing assistants on a working contract at the selected units, no specific exclusion criteria were identified. The survey was accessible through a direct link and all data was collected anonymously, using the system Webropol. Before the survey started, the aim and content of the study was described and consent was obtained. After submitting the questionnaire, no withdrawal of participation was possible. Two email reminders were sent out two and four weeks following the initial email. Potential participants thereby had a total of four weeks to take part in the study.

### Data analysis

Means and standard deviations were used to describe continuous variables and frequency counts and percentages for categorical variables. Normality was assessed using Shapiro-Wilk test and Q-Q plots. Independent t-test was used for continuous variables with normal distribution and Mann Whitney U-test for non-normally distributed data. Fisher’s exact test was used for categorical variables. Data from the web-based survey was extracted and entered into SPSS for Mac, version 26 (SPSS Inc., Chicago, Illinois, USA). Significance level for all tests was set at *p* < 0.05. Free-text answers were read and reviewed to get a general sense of the data. They were then coded and lemmatized to group similar descriptions of symptoms using text analysis [29]. For each group, topic analysis was applied to define a set of keywords relating to each symptom category. Due to the brevity of the open-ended responses, results were presented with a quote to exemplify each symptom category [29].

## Results

### Participant characteristics

**Fig. 1:**
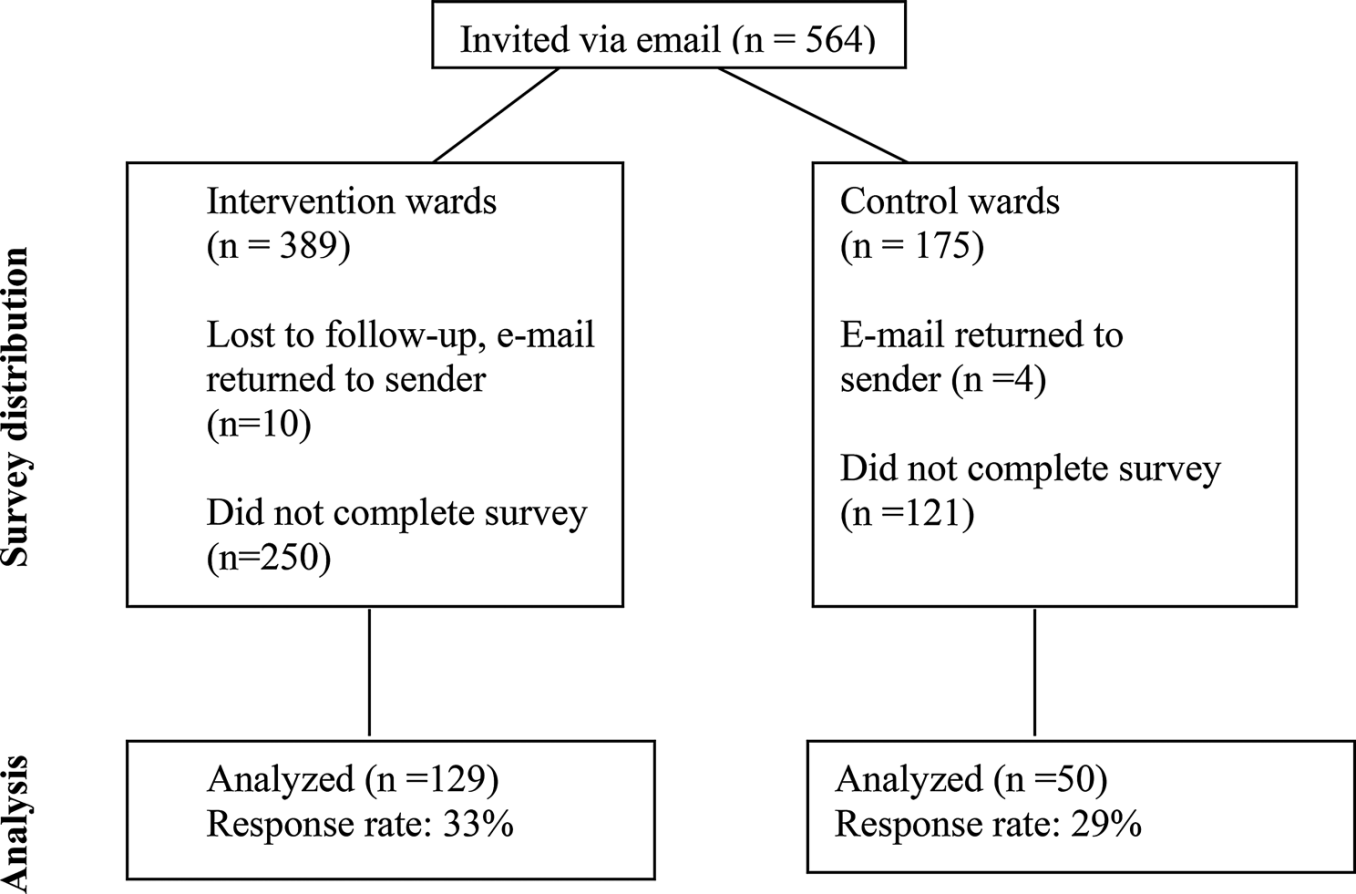
CONSORT diagram illustrating the survey distribution work flow

Out of 564 initially invited to participate, 179 completed the questionnaires. Of these, 129 were from the intervention units and 50 from the control units yielding a total response rate of 32% (Fig. 1). Respondents from the intervention units were significantly younger than their counterparts at the control units (42.9 years vs. 48.5 years respectively, *p* = 0.011). The majority (81%) were female. Roughly half were registered nurses and the remaining worked as nursing assistants (Table 1). The professional experience was similar between the groups, as well as the distribution of respondents working day, night or combined shifts. The questions and items in web-based questionnaire were not compulsory, and respondents skipped occasional questions. All respondents provided baseline characteristics but the number of respondents to each part of the subsequent questionnaires are provided in the table headings (Tables 2, 3 and 4).

**Table 1:** Characteristics of the study participants

	Intervention units (n=129)	Control units (n=50)	*P*
Age (years)	42.9 (13.01)	48.5 (12.66)	0.011*
**Sex**			0.570
Male	23 (18)	6 (12)	
Female	102 (79)	43 (86)	
Did not specify	4 (3)	1 (2)	
**Unit type**			
Heart or vascular	46 (36)	0	N/A
Neurology or neurosurgery	82 (64)	0	
Ear-nose-throat surgery	0	10 (20)	
Breast/endocrine oncological surgery	0	9 (18)	
Pancreatic/liver oncologic surgery	0	16 (32)	
Urology	0	15 (30)	
Did not specify	1 (1)	0	
**Professional role**			
Registered nurse	60 (47)	28 (56)	0.179
Nurse assistant	69 (53)	20 (44)	
Did not specify	0	2 (4)	
**Highest academic degree for RNs**			
Bachelor	48 (81)	24 (86)	0.431
Master degree or licentiate	11 (19)	4 (14)	
Experience in professional role (years)	13.48 (11.11)	16.40 (11.49)	0.135
Experience at current unit (years)	8.58 (8.68)	6.42 (6.42)	0.079
**Predominant shift work**			0.844
Day-shift only	73 (57)	27 (54)	
Night-shift only	34 (27)	15 (30)	
Combined day and night	21 (16)	7 (14)	
Did not specify	1 (1)	1 (2)	

SD: Standard deviation; RN: Registered nurse. Continuous, normally distributed variables are expressed as mean (standard deviation) and categorical data are presented as counts and percentage (n, %)

### Values, knowledge and experiences of patients’ sleep

On a scale of 0–10, sleep was rated as highly important during in-hospital care among respondents at both the intervention and control units (9.16 vs. 9.20 respectively, *p* = 0.724) (Table 2). There was no difference between the groups regarding neither self-rated knowledge of sleep nor knowledge of sleep-promoting activities. A majority of the respondents in both groups wanted to increase their knowledge of sleep promotion (75% in the intervention group and 80% in the control group, *p* = 0.692). Experiences of situations where insufficient sleep had affected patients negatively were described by 91% in the intervention group and 94% in the control group (*p* = 0.741). Consequences of insufficient sleep were described in open-ended format and are presented in Table 2. The most frequently reported consequences of insufficient sleep were psychological symptoms (20%), followed by fatigue (16%), disturbed sleep pattern (15%) and immobilisation (15%). Respondents described the consequences of inadequate sleep as a cascade of symptoms, deteriorating the patients’ chances of recovery and recuperation. Participants described that as patients’ physical and psychological symptoms were amplified by sleep deprivation, it negatively affected their ability to participate in nursing activities such as mobilisation and eating.

**Table 2:** Values, knowledge and experiences of patients’ sleep

	Intervention units (*n* = 128)		Control units (*n* = 50)		*P*
Importance of sleep during in-hospital care, mean (SD)	9.16 (1.315)		9.20 (1.384)		0.724
Self-rated knowledge of sleep, mean (SD)	6.24 (2.042)		6.13 (2.330)		0.853
Self-rated knowledge of sleep-promoting activities, mean (SD)	6.41 (1.988)		6.29 (2.259)		0.956

	Yes	No	Yes	No	P
Would you like to increase your knowledge of sleep promotion? n (%)	95 (75)	31 (25)	39 (80)	10 (20)	0.692

	Yes	No	Yes	No	P
Do you have experience of situations where insufficient sleep has affected patients negatively?	116 (91)	12 (9)	47 (94)	3 (6)	0.741

Symptom categories of insufficient sleep	Frequency, n (%)		Quote		
Pain	15 (8)		“They have an increased sensitivity to pain.”		
Fatigue	29 (16)		“Patients can be really tired in the day and unable to cooperate.”		
Nutrition	18 (10)		“They’re missing out on meals during the day because they are sleeping instead.”		
Immobilization	27 (15)		“Patients are too tired to be mobilized in the day because they are awakened several times at night for medical treatments.”		
Reduced participation	10 (6)		“They get tired and don’t have the energy to be active, everything becomes a struggle and symptoms are amplified when the patient hasn’t slept.”		
Psychological symptoms	36 (20)		“Patients experience more worry, pain and some state feeling “crazy” when they don’t get to sleep.”		
Delirium	20 (11)		“They get confused, motorically anxious, develop delirium.”		
Disturbed sleep pattern	27 (15)		“They flip their days, the staff thinks it’s good if they rest but instead they create an unhealthy sleep pattern.”		

### Non-pharmacological methods of enhancing sleep

Participants used various non-pharmacological methods to minimise disruption and enhance patients’ sleep in hospital, and the methods were similarly distributed between the intervention and control units (Table 3). The most common non-pharmacological methods were adjusted lighting, and adjusted timing of examinations, blood draws and medicine administrations.

**Table 3:** Non-pharmacological sleep-enhancing methods currently used at the intervention and control units respectively

	Intervention units (*n* = 128)	Control units (*n* = 50)	*P*
Adjusted lighting	119 (94)	45 (90)	0.522
Ear plugs	30 (24)	12 (26)	0.441
Eye mask	3 (2)	0	0.564
Adjusted examinations	67 (52)	28 (56)	0.739
Adjusted blood draws	58 (45)	20 (41)	0.735
Adjusted medicine administrations	65 (50)	25 (50)	1.000

Categorical data are presented as counts and percentage (n, %)

### Attention given to sleep during hospitalization

There were no significant differences in the attention given to sleep during the hospitalisation between the intervention and control units (Table 4). Two thirds of the respondents in both groups stated that they addressed sleep when admitting patients to hospital, and a similar proportion included sleep at their end-of-shift reports. A minority (39% in the intervention units and 30% in the control units, *p* = 0.263) included sleep as a subject when patients were transferred between units, and only ¼ stated that sleep medications were evaluated at the medical rounds.

**Table 4:** Attention given to sleep during the care pathway, presented as numbers and proportions of respondents stating that they always or often perform the following sleep-related activities

	Intervention units (*n* = 124)	Control units (*n* = 50)	*P*
Sleep addressed at admission	76 (62)	34 (68)	0.479
Sleep documented at admission	58 (46)	27 (55)	0.281
Sleep addressed at transfer	48 (39)	15 (30)	0.263
Sleep addressed at ward rounds	59 (47)	20 (40)	0.387
Sleep addressed at shift change	84 (68)	35 (70)	0.772
Sleep documented continuously during hospital stay	59 (46)	28 (56)	0.253
Sleep medications evaluated at the ward round	35 (28)	12 (24)	0.550
Sleep addressed at discharge	37 (30)	16 (32)	0.779

Categorical data are presented as counts and percentage (n, %)

### Implementation and uptake of sleep-promoting intervention

Specific questions regarding the sleep-promoting intervention were administered to respondents at the intervention units to assess the uptake of the intervention among nursing staff (Table 5). These questions were completed by 128/129 respondents (99%). The web-based sleep course was known to 53% (*n* = 68) of the respondents. Of those who knew of the course, 48% (*n* = 32) had completed the course and 84% (*n* = 27) stated that they had gained new or deepened knowledge. Regarding the clinical guideline on in-hospital sleep, 42 respondents (33%) were aware of the guideline, 41% of these had read the guideline and 90% of those stated that it had provided them with new or deepened knowledge. When surveying the uptake of the SNC role, 46 respondents (36%) knew that there was a SNC at their unit. Of these, 20 (44%) had consulted the SNC for advice and guidance on patients’ sleep. Irrespective of having consulted the SNC for specific guidance, 32 respondents (69%) stated that the SNC had contributed with new knowledge about sleep and sleep promotion in their ward unit.

**Table 5:** Uptake of the three components of a sleep-promoting intervention among nursing staff at the intervention units

	Intervention group (*n* = 128)		
**Question 1 – Web-based course**	Yes		No
Are you aware that there is a web-based sleep course?	68 (53)		60 (47)
**If yes**, have you completed the web-based course?	Yes	No	
	32 (48)	35 (52)	
**If yes**, did it provide you with new or deepened knowledge?	Yes	No	
	27 (84)	5 (16)	
**Question 2 – Clinical guideline**	Yes		No
Are you aware that there is a clinical guideline on in-hospital sleep?	42 (33)		84 (67)
**If yes**, have you read the guideline?	Yes	No	
	18 (41)	26 (59)	
**If yes**, did it provide you with new or deepened knowledge?	Yes	No	
	18 (100)	0	
**Question 3 – Sleep nursing champion (SNC)**	Yes		No
Is there a SNC at your unit?	46 (36)		81 (64)
**If yes**, have you consulted him/her regarding patients’ sleep?	Yes	No	
	20 (44)	25 (56)	
Has the SNC contributed with new knowledge in your unit?	Yes	No	
	32 (69)	14 (31)	

SNC: Sleep nursing champion. Categorical data are presented as counts and percentage (n, %).

## Discussion

This study aimed to describe the knowledge and values of evidence-based sleep promotion among nursing staff working with hospitalised patients. A secondary aim was to assess the attention given to sleep promotion and implementation of a sleep-promoting intervention. Across both groups, respondents rated sleep as highly important during in-hospital care, and exhibited extensive experience of situations where patients’ health had been negatively affected by impaired sleep. The sleep-enhancing methods used were mainly adjusted lighting, and adjusted timing of examinations and medicine administrations. Sleep was addressed upon admission and end-of-shift reports, but less frequently addressed and evaluated at ward rounds or discharge. In the current study, patients’ perceptions of in-hospital sleep were not charted. However, across both groups, 93% reported having experience of situations where insufficient sleep has affected patients negatively and a wide range of symptoms and examples were given. Various non-pharmacological methods were used to minimize disruption and enhance patients’ sleep in hospital, and the methods were similarly distributed between the intervention and control units. The most commonly used non-pharmacological methods, adjusted lighting and timing of procedures reflect basic environmental modifications rather than structured interventions. Additionally, certain non-pharmacological methods to promote in-hospital sleep such as music and relaxation therapy were not charted in the current study. The use of these methods in the current setting can therefore not be assessed. A network meta-analysis concluded that sleep-promoting nursing interventions adopt a wide variety of different elements, but that a combination of eye masks and ear plugs stood out as the most effective interventions to enhance sleep quality in critically ill patients [30]. To evaluate evidence-based sleep interventions, a demarcation of the range of nursing activities and elements associated with sleep promotion is warranted.

On a scale of 0–10, sleep was rated as highly important during in-hospital care among respondents at both the intervention and control units. Hence, the motivation to enhance patients’ sleep seems to be high, irrespective of the intervention, which is in line with an integrative review, stating that organisational policies and coworkers’ attitudes delimit the implementation of sleep promoting interventions [18]. In our results, sleep was inconsistently addressed during patient transitions and medical rounds, indicating that sleep remains marginal in clinical communication and decision-making processes. Sleep-derangements during hospitalisation include noise disturbance and operational interruptions [31–34], largely induced by health care staff and resulting in a worsening of sleep following hospitalisation [35]. Restructuring the clinical workflow to minimise night time disruption has proven effective in improving patients’ sleep [36], and addressing sleep in clinical interprofessional settings such as medical rounds may be an important contribution to this awareness.

Throughout the results, no significant differences were seen between the intervention and control units. Furthermore, only 53% of the respondents at the intervention units were aware of the web-based course. In terms of the clinical guidelines and SNCs, corresponding numbers were even lower (33% and 36% respectively). The lack of significant differences between intervention and control groups regarding knowledge, attention to sleep, and use of non-pharmacological methods suggests that awareness alone may not be sufficient to drive behavioural change. Although the intervention included a web-based course, clinical guidelines, and designated sleep nursing champions (SNCs), only a minority of staff were aware of or had engaged with these resources. This gap between the availability of sleep-promoting tools and their practical implementation in clinical routines may be due to the lack of active dissemination strategies in the current intervention. While many respondents expressed a desire to increase their knowledge of sleep promotion, this motivation did not translate into widespread participation in the intervention activities. These findings underscore the need for a more robust implementation strategy that actively involves healthcare staff and patients. Successful integration of sleep-promoting practices requires not only educational resources but also organizational support, leadership engagement, and reinforcement through clinical routines [37].

The implementation of complex nursing interventions in clinical practice requires patience and persistence, and there are numerous strategies and models to aid implementation of evidence-based methods in healthcare. The current study utilized the elements of the i-PARIHS framework, which specifies determinants that act as enablers or barriers influencing implementation outcomes [38]. Within this framework, a task-oriented approach to implementation is applied [20]. One key aspect to successful implementation is facilitation, i.e. promoting change through engagement, understanding and negotiation [39]. Although the SNCs in the current project were provided with ample support by the research group, no detailed plan for implementation was set up. Training of nurses as well as implementation of sleep policies and frameworks have all been suggested as means of implementing sleep hygiene interventions in the emergency care setting [32, 40]. The dissemination of evidence-based nursing practices in clinical settings through local coaches is a well-established strategy but warrants small, clearly outlined steps to attain lasting effects in the local learning culture [41]. It is plausible that these steps were inadequately outlined in the current study. Clear goal setting and resource allocation from key stakeholders are vital aspects of implementation [39], but these aspects were not considered in the current study. Our results therefore provide an indication of the current implementation status, rather than finite results of an implemented sleep-promoting intervention.

### Strengths and limitations of the study

The cross-sectional, single center study design has inherent limitations as it fails to assess the implementation of the intervention over time or across multiple hospitals. It thereby limits the transferability of the results. Furthermore, the effects of the intervention on patients’ sleep quality has not been reported and is yet to be assessed. The high interest and value reported in the current study may pertain to acquiescence bias, but may also be a result of response bias where those with an interest in sleep promotion may be more prone to take the survey. Whether this interest translates into practice is yet to be determined. The findings are limited by the restricted sample. The response rate was 32% and while web-based questionnaires yield approximately 12–15% lower response rates than paper questionnaires [42], they were chosen due to their cost-effectiveness and practical superiority. Reminders were sent out to maximise participation. The low response rate in the current study is in line with those previously reported but still challenges the validity of our findings [43]. It is plausible that a higher response rate may have altered the findings. Furthermore, validity is challenged by the web-based questionnaire. It was developed by author LG and has been used in a previous study but has not been psychometrically evaluated.

## Conclusion

Nursing staff are dedicated to sleep promotion and wish to increase their knowledge of non-pharmacological methods to promote sleep during hospitalization. However, the uptake of a sleep-promoting intervention in an acute care setting was limited and the staff did not utilize the available tools and resources. For successful dissemination of evidence-based sleep-promotion, active implementation strategies utilizing the engagement of patients, healthcare staff and policy makers are crucial.

## Data Availability

All study data was fully available to the authors, maintaining the integrity of the data and accuracy of the data analysis. The authors have checked to make sure that our submission conforms as applicable to the Journal’s statistical guidelines. All statistical analyses were performed by the first author. The authors affirm that the methods used in the data analyses are suitably applied to their data within their study design and context, and that statistical findings have been implemented and interpreted correctly. The authors agree to take responsibility for ensuring that the choice of statistical approach is appropriate and is conducted and interpreted correctly as a condition to submit to the Journal.
